# Nutrient Content of Squeeze Pouch Foods for Infants and Toddlers Sold in the United States in 2015 †

**DOI:** 10.3390/nu11071689

**Published:** 2019-07-23

**Authors:** Jennifer L. Beauregard, Marlana Bates, Mary E. Cogswell, Jennifer M. Nelson, Heather C. Hamner

**Affiliations:** 1 Division of Nutrition, Physical Activity, and Obesity, National Center for Chronic Disease Prevention and Health Promotion, Centers for Disease Control and Prevention, Atlanta, GA 30341-3717, USA; 2 U.S. Public Health Service Commissioned Corps, Rockville, MD 20852, USA; 3 Epidemic Intelligence Service, Center for Surveillance, Epidemiology, and Laboratory Services, Centers for Disease Control and Prevention, Atlanta, GA 30341-3717, USA; 4 Division for Heart Disease and Stroke Prevention, National Center for Chronic Disease Prevention and Health Promotion, Centers for Disease Control and Prevention, Atlanta, GA 30341-3717, USA; 5 Oak Ridge Institute for Science and Education (ORISE), Oak Ridge, TN 37831-0117, USA

**Keywords:** infant, toddler, nutrition, complementary feeding

## Abstract

Background: To describe the availability and nutrient composition of U.S. commercially available squeeze pouch infant and toddler foods in 2015. Materials and Methods: Data were from information presented on nutrition labels for 703 ready-to-serve, pureed food products from 24 major U.S. infant and toddler food brands. We described nutritional components (e.g., calories, fat) and compared them between packaging types (squeeze pouch versus other packaging types) within food categories. Results: 397 (56%) of the analyzed food products were packaged as squeeze pouches. Differences in 13 nutritional components between squeeze pouch versus other packaging types were generally small and varied by food category. Squeeze pouches in the fruits and vegetables, fruit-based, and vegetable-based categories were more likely to contain added sugars than other package types. Conclusion: In 2015, squeeze pouches were prevalent in the U.S. commercial infant and toddler food market. Nutrient composition differed between squeeze pouches and other packaging types for some macro- and micronutrients. Although it is recommended that infants and toddlers under two years old not consume any added sugars, a specific area of concern may be the inclusion of sources of added sugar in squeeze pouches. Linking this information with children’s dietary intake would facilitate understanding how these differences affect overall diet quality.

## 1. Introduction

Early eating environments are associated with children’s later dietary patterns and may be shaped by a number of influences including parents’ or caregivers’ own eating behaviors and food selections, as well as the choices that they make in terms of which foods to offer and how to introduce them to young children [1]. A recent development in the global commercial infant and toddler food market is the introduction of squeeze pouch foods. Squeeze pouch foods were introduced into the U.S. commercial infant and toddler food market in 2008 and now account for a quarter of U.S. baby food sales [2]. In 2015, the United States had the largest squeeze pouch market globally with sales of squeeze pouches growing while sales of products sold in traditional glass or plastic packaging remaining stable [3]. Parents or caregivers may choose to offer squeeze pouches for convenience, less mess, and allowing children to feed themselves independently [4]. However, feeding with squeeze pouches can lack the sensory experience of seeing, smelling, and touching new foods and result in less time devoted to sitting down and feeding, which may be important for modeling of eating behaviors [1]. 

News articles [2], Internet blogs [5], and health organizations [6] have noted benefits of and concerns about squeeze pouch foods. Recently, several perspective pieces in scientific journals noted concerns about infants and toddlers consuming foods out of squeeze pouches due to their potential impacts on infants’ and children’s eating behaviors [7,8,9] as well as their nutrient composition [7,8]. Two of these articles analyzed the nutrient composition of a select sample of squeeze pouch products sold in Germany (*n* = 100) [7] and Denmark (*n* = 10) [8] and found that high sugar content was a specific area of concern. To our knowledge, there has been no published report of the nutritional contents of squeeze pouch foods sold in the United States, and no report has examined the full range of products on the market. Several recent studies described the nutrient composition of the broader range of commercial infant and toddler foods sold in the United States [10,11,12], but none specifically examined the contents of squeeze pouch foods. 

The objective of this study was to describe the availability and nutrient composition (based on information presented on nutrition labels) of commercially available squeeze pouch infant and toddler foods in the United States. These products were also compared with other pureed, ready-to-serve foods available in other packaging types (e.g., glass jars and plastic packs) in order to determine whether there were differences in the nutrient composition of pureed, ready-to-serve foods by packaging type. We hypothesized that the nutrient content of commercially available squeeze pouches would differ compared with other pureed, ready-to-serve foods available in other packaging types (e.g., glass jars and plastic packs). We were specifically interested in understanding whether or not the presence of added sugar differed by packaging type. Given that added sugar is not recommended for children under two years old in the United States [13] but other studies have found higher amounts of added sugar in the broader context of U.S. infant and toddler foods [10,12], this was of particular interest.

## 2. Materials and Methods

Our data source comprised 1,037 commercial infant and toddler food products from 24 major brands of infant and toddler foods available in the United States in 2015. These brands account for >95% of market share in U.S. infant and toddler food sales [3]. Methods for selecting brands and identifying products have been previously described [10]. Briefly, visits were made to nine retail and wholesale grocers and two drugstores in metropolitan Atlanta, Georgia and Seattle, Washington in May-July 2015 to identify infant and toddler food products. Infant formulas, fortified milk, and oral electrolytes were excluded because the U.S. Food and Drug Administration regulates nutritional content and labeling for these products separately [14]. Nutritional data were obtained from manufacturers’ website when available (*n* = 934), in-store (*n* = 82), or from both sources (*n* = 21) in cases where we found partial information online or in-store and label data were completed using the other source.

Of the 1037 products, 703 products consisting of pureed, ready-to-serve foods were eligible for our analyses (Table S1). Pureed was defined as not requiring chewing; the consistency of these products varied with age (thinner for products intended for younger consumers, thicker for products intended for older consumers). Ready-to-serve was defined as not requiring additional preparation, such as adding water. Yogurt-containing products were included when similar in texture to other types of purees (e.g., fruit purees); yogurt-containing drinks and freeze-dried snacks were excluded. We excluded drinks as well as products that contained small soft pieces of food because these did not meet our definition of pureed, ready-to-serve foods. 

We identified the container type of each product using previously defined categories: [10] bag, box, can, glass jar, juice box, plastic bottle, plastic bowl, plastic box, plastic container, plastic pack, pouch, squeeze pouch, and tray (Table S1). For this analysis, we dichotomized the 703 eligible products by packaging type into squeeze pouches (defined as being re-sealable, squeezable, and having a twist-off cap) versus all other packaging types. Then, we classified products by stage of targeted eater (using previously classified categories [10]), and by brand. Stages (age of targeted eater) may vary by brand but are generally defined as stage 1 (~4–6 months), stage 2 (~7–8 months), stage 3 (~9–12 months), and stage 4 (~12 months and older).

We classified the 703 eligible products into five mutually exclusive food categories (meat-, fish-, or legume-based; yogurt- or milk-based; fruits and vegetables; fruit-based; and vegetable-based) based on the full ingredient list of the product. The lead author (JB) developed the product classification scheme (see Web Supplement). Two authors (JB, MB) independently reviewed each product’s full ingredient list and assigned it a food category. Discrepancies in coding were identified (*n* = 7) and resolved via discussion (see Web Supplement). 

Nutritional components of interest were serving size (g), calories (per 100 g), fat (g per 100 g), protein (g per 100 g), total carbohydrates (g per 100 g), fiber (g per 100 g), sugar (g per 100 g), sodium (mg per 100 g), vitamin A (mcg per 100 g), vitamin C (mg per 100 g), calcium (mg per 100 g), and iron (mg per 100 g). These nutrient components were analyzed as continuous outcome variables in our analyses. We also created a binary indicator for whether or not a product contained ≥1 source of added sugars (e.g., sugar, sweetener, syrup, juice concentrate) based on the method developed by Maalouf et al. (2017) [10]. Juice concentrate was not considered an added sugar when combined with water to reconstitute to a single strength. There were 21 products missing data on vitamin A, vitamin C, calcium, and iron, as well as one product missing data on calcium only. These products were excluded from analyses of those specific vitamins and minerals. All products had complete data on the other nutritional components.

We stratified all analyses by food category and packaging type. Within each group, we calculated the median and interquartile range (IQR) for each continuous outcome variable (i.e., all outcome variables except for whether the product contained added sugars) because the distributions of many of the outcome variables were skewed after stratifying by food category and packaging type. Wilcoxon rank–sum tests were used to test whether the continuous outcome variables differed between squeeze pouches and other packaging types; *p*-values were corrected for multiple testing using the Benjamini–Hochberg procedure [15]. We calculated the percentage (and 95% binomial confidence interval) of products containing added sugars. Chi-square tests assessed whether added sugar content differed between squeeze pouches and other packaging types, using an alpha level of 0.05.

Of the 18 brands that included pureed, ready-to-serve products, six brands included both squeeze pouches and other packaging types. In a sensitivity analysis, we restricted analyses to brands that included both squeeze pouches and other packaging types (*n* = 466 across six brands) in order to assess whether differences in nutrient composition between packaging types could be driven by differences in nutrient composition across brands. 

Analyses were conducted using SAS version 9.4 (Cary, NC, USA). This study was not reviewed by an institutional review board because it did not involve research on human subjects. All data were publically available.

## 3. Results

Among the 703 products containing pureed, ready-to-serve foods, 397 (56%) were squeeze pouches and 306 (44%) were other packaging types (Table 1). Of the 306 products in other packaging types, 69% were in glass jars, 23% in plastic packs, 6% in plastic containers, and <1% in non-squeezable pouches. 

**Figure 1:**
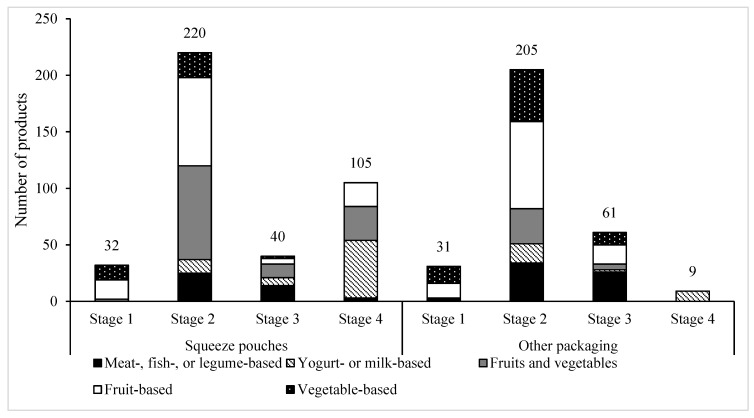
Pureed, ready-to-serve products by packaging type, stage, and food category (*n* = 703). Approximate age of targeted eater by stage are as follows: Stage 1 (~4–6 months), stage 2 (~7–8 months), stage 3 (~9–12 months), stage 4 (~12 months and older).

Food categories and intended stage of eater differed between squeeze pouches and other packaging types (Figure 1). Two-thirds of squeeze pouches were fruit-based or fruit- and vegetable-based compared with less than half of other packaging. Less than 10% of squeeze pouches were vegetable-based compared with nearly a quarter of other packaging types. The majority of products were targeted at stage 2 eaters (55% of squeeze pouch products, 67% of other packaging types). However, 26% of squeeze pouch products were targeted at stage 4 eaters compared with 3% of other packaging types. The majority of squeeze pouches for stage 4 eaters consisted of yogurt- or milk-based products.

Differences in nutrient composition between squeeze pouches and other packaging types varied across food categories. These differences were generally small in magnitude (Table 2). Statistically significant findings are highlighted. Among meat-, fish-, or legume-based products, squeeze pouches contained fewer calories and less fat but more fiber, vitamin C, calcium, and iron compared with other packaging types. Among yogurt- or milk-based products, squeeze pouches contained fewer calories and less fat, protein, sodium, and calcium but more fiber, vitamin C, and iron compared with other packaging types. Among fruit and vegetable products, squeeze pouches were larger than other packaging types, contained more protein and vitamin C, and were more likely to contain added sugar compared with other packaging types. Among fruit-based products, squeeze pouches contained more sugar and were more likely to contain added sugar but less sodium compared with other packaging types. Finally, among vegetable-based products, squeeze pouches contained more vitamin C and were more likely to contain added sugar compared with other packaging types.

After restricting to brands that produced both squeeze pouches and other packaging types, medians and IQRs were similar, but there were some differences in patterns of statistical significance of comparisons (Table S2). 

## 4. Discussion

We found select statistically significant differences in nutritional components between squeeze pouches and other packaging, but many of the observed differences were small in magnitude. Although it is recommended that infants and toddlers under two years old not consume any added sugars [13], squeeze pouches in the fruits and vegetables, fruit-based, and vegetable-based categories were more likely to contain added sugars than other package types. Our findings echo those of previous studies noting high sugar content in commercial infant and toddler foods [12] in the United States and in select squeeze pouch foods sold in Germany [7] and Denmark [8]. A specific area of concern may be the inclusion of sources of added sugar in squeeze pouches.

Although most of the differences in nutritional components between squeeze pouches and other packaging were small in magnitude, further study is required of the impacts of the use of squeeze pouch products on infant’s and children’s early eating environments and how these may affect their later dietary patterns [1]. Squeeze pouches can lack the sensory experiences of consuming new foods and result in less time devoted to sitting down and feeding. These experiences may be important for modeling of eating behaviors [1]. Further, it is concerning that one-quarter of squeeze pouches analyzed in our study were targeted at stage 4 eaters (~12 months and older). A recent U.S. expert panel of pediatricians and nutrition scientists called for children to fully transition to consuming the family diet by two years of age [1], earlier exposure to a variety of textured foods in the first year (e.g., mashed, lumpy, or chopped rather than pureed) supports the transition to consuming family foods [16,17,18,19].

Our findings on added sugar content of squeeze pouches in certain food categories are in line with findings from two recent articles on limited samples of squeeze pouches sold in Germany (*n* = 100) [7] and Denmark (*n* = 10) [8], which noted high sugar content and high percentage of energy from sugar of 80% or higher. The authors of these papers (a consensus paper from the Nutrition Commission of the German Society for Pediatrics and Adolescent Medicine [7] and an editorial from members of the World Health Organization [8]) concluded that pureed or liquid foods in plastic pouches should not be regularly consumed by infants and young children due to their high energy density and high sugar content. They also noted that squeeze pouches limit the opportunity for spoon and hand feeding. Our study builds upon these findings by assessing a broader range of squeeze pouch products of all food categories, by assessing products sold in the United States since results may not be generalizable across the international food supply, and by comparing the nutrient content of pureed foods in squeeze pouches to those in traditional packaging. 

Continued assessment of commercial infant and toddler food products—including a focus on the mode of feeding (e.g., squeeze pouches vs. traditional jarred products)—could help improve the understanding of the relationship between dietary practices and dietary quality. For example, these analyses could yield information on how much of toddlers’ (i.e., 12 months and older) diets are made up of squeeze pouches, given that one-quarter of squeeze pouches are targeted to this age group. One potential source of data could be the Nestlé Feeding Infants and Toddlers Study (FITS), a cross-sectional survey of caregivers of U.S. infants, toddlers, and preschool children conducted in 2002, 2008, and 2016. A new question on the use of food from pouches was added to the 2016 FITS survey. To our knowledge, FITS data on squeeze pouches have not yet been published.

Our study has several limitations and strengths. Nutritional data were obtained from product labels and were not independently verified by the authors. U.S. federal law governing nutrition labeling permits declared nutrient values on product labels to differ from actual values by up to 20% [20]. It is possible that labeled nutrient values could differ from actual values. Our analyses did not account for differences in market share of individual products. While our study is strengthened by the robust search strategy used to identify products, it is possible that some products available in the market were missed (e.g., private-label brands available in regions of the country other than those surveyed). However, the selected brands represent over 95% of U.S. baby food market share, and we obtained the full product line for each of these brands from their websites. Because of the changing market place, products that were available in 2015 may no longer be available or newer products may now be present. However, the strength of this study is that it provides new information on differences in the nutritional composition of commercial infant and toddler foods by packaging type, assessment of added sugar content using information from product ingredient lists, and a description of the number of brands offering products in squeeze pouch packaging. 

## 5. Conclusions

In 2015, squeeze pouches comprised the majority of pureed, ready-to-serve commercial infant and toddler food products sold in the United States. Food categories of pureed, ready-to-serve products differed by squeeze pouch versus other packaging types, and squeeze pouches more often targeted later stage eaters than other packaging types. Comparing squeeze pouches versus other packaging types, nutrient composition differed for some macro- and micronutrients. Despite clinical recommendations that infants and toddlers under two years old not consume any added sugars, squeeze pouch products were significantly more likely to contain added sugars. Linking information on nutrient composition of squeeze pouch products with children’s dietary intake would facilitate understanding how these differences in macro- and micronutrients affect overall diet quality.

## Figures and Tables

**Table 1:** Characteristics of pureed, ready-to-serve products, by packaging type (*n* = 703).

Characteristic	Squeeze Pouch		Other Packaging	
	*n*	% ^a^	*n*	% ^a^
Total	397	100%	306	100%
Food category				
Meat-, fish-, or legume-based	42	11%	63	21%
Yogurt- or milk-based	70	18%	28	9%
Fruits and vegetables	127	32%	36	12%
Fruit-based	121	30%	107	35%
Vegetable-based	37	9%	72	24%
Stage				
1	32	8%	31	10%
2	220	55%	205	67%
3	40	10%	61	20%
4	105	26%	9	3%

^a^ Percentages may not add to 100 due to rounding. Approximate age of targeted eater by stage are as follows: Stage 1 (~4–6 months), stage 2 (~7–8 months), stage 3 (~9–12 months), stage 4 (~12 months and older).

**Table 2:** Comparison of macro- and micronutrient composition between squeeze pouch and other packaging (*n* = 703).

Nutritional Component	Squeeze Pouch		Other Packaging		*p*
	Median	(IQR)	Median	(IQR)	
Meat-, fish-, or legume-based					
Serving size, g	113.0	(99.0–127.0)	113.0	(99.0–170.0)	0.2408
Calories (Kcal/100 g)	61.9	(53.1–70.8)	70.7	(61.9–88.2)	0.0053 *
Fat (g/100 g)	1.0	(0.4–2.0)	1.8	(0.9–3.0)	0.0005 *
Protein (g/100 g)	2.7	(1.8–3.1)	2.4	(1.8–3.2)	0.7352
Carbohydrates (g/100 g)	9.9	(8.8–12.4)	10.1	(8.0–12.9)	0.6259
Fiber (g/100 g)	2.0	(1.6–2.7)	1.1	(0.6–1.8)	<0.0001 *
Sugar (g/100 g)	2.7	(1.8–3.9)	2.9	(1.7–4.0)	0.7284
Sodium (mg/100 g)	17.3	(8.9–27.6)	29.4	(16.7–39.8)	0.0270
Vitamin A (mcg/100 g)	1084.1	(413.4–1858.4)	796.5	(50.0–1991.2)	0.1582
Vitamin C (mcg/100 g)	0.7	(0.0–1.9)	0.0	(0.0–0.6)	0.0048 *
Calcium (mg/100 g)	21.2	(12.1–36.4)	12.4	(10.6–21.2)	0.0002 *
Iron (mg/100 g)	0.6	(0.5–0.9)	0.5	(0.3–0.7)	0.0073 *
Contained added sugar (%) ^1^	4.8%	(0.6–16.2%)	3.2%	(0.4–11.0%)	0.6772
Yogurt- or milk-based					
Serving size, g	113.0	(99.0–120.0)	113.0	(99.0–113.0)	0.0249
Calories (Kcal/100 g)	79.6	(66.7–93.5)	97.3	(88.5–101.0)	0.0004 *
Fat (g/100 g)	0.9	(0.4–1.7)	3.0	(3.0–3.5)	<0.0001 *
Protein (g/100 g)	1.9	(0.9–2.7)	3.0	(3.0–3.5)	<0.0001 *
Carbohydrates (g/100 g)	14.3	(13.3–17.5)	14.7	(12.4–16.2)	0.4812
Fiber (g/100 g)	1.1	(0.8–1.8)	0.0	(0.0–0.4)	<0.0001 *
Sugar (g/100 g)	11.1	(8.9–12.9)	11.5	(10.6–12.1)	0.5815
Sodium (mg/100 g)	17.2	(8.9–26.6)	53.1	(47.4–55.6)	<0.0001 *
Vitamin A (mcg/100 g)	106.7	(30.3–312.5)	213.2	(132.7–239.9)	0.1002
Vitamin C (mcg/100 g)	8.3	(1.9–16.8)	0.0	(0.0–0.0)	<0.0001 *
Calcium (mg/100 g)	59.8	(28.3–133.3)	106.2	(90.9–132.7)	0.0108 *
Iron (mg/100 g)	0.3	(0.0–0.4)	0.0	(0.0–0.0)	<0.0001 *
Contained added sugar (%) ^1^	70.0%	(57.9–80.4%)	78.6%	(59.0–91.7%)	0.3909
Fruits and vegetables					
Serving size, g	113.0	(107.0–120.0)	60.0	(60.0–113.0)	<0.0001 *
Calories (Kcal/100 g)	61.9	(53.1–70.7)	58.3	(50.0–64.3)	0.0190
Fat (g/100 g)	0.0	(0.0–0.0)	0.0	(0.0–0.0)	0.4714
Protein (g/100 g)	0.8	(0.4–0.9)	0.0	(0.0–0.4)	<0.0001*
Carbohydrates (g/100 g)	14.1	(12.4–15.9)	13.3	(11.4–13.3)	0.0238
Fiber (g/100 g)	1.8	(1.0–2.5)	1.7	(1.7–2.5)	0.8420
Sugar (g/100 g)	9.7	(8.0–10.8)	8.3	(6.7–10.0)	0.0323
Sodium (mg/100 g)	8.9	(4.2–16.7)	2.2	(0.0–16.7)	0.0262
Vitamin A (mcg/100 g)	464.6	(132.7–1327.4)	615.5	(50.0–1413.7)	0.9458
Vitamin C (mcg/100 g)	13.1	(2.5–20.0)	0.0	(0.0–1.0)	<0.0001 *
Calcium (mg/100 g)	10.6	(9.6–21.2)	20.0	(5.0–20.0)	0.2539
Iron (mg/100 g)	0.3	(0.3–0.5)	0.5	(0.3–0.5)	0.4770
Contained added sugar (%) ^1^	57.5%	(48.4%–66.2%)	33.3%	(18.6%–51.0%)	0.0105†
Fruit-based					
Serving size, g	99.0	(90.0–113.0)	113.0	(99.0–113.0)	0.2073
Calories (Kcal/100 g)	66.7	(60.6–79.6)	64.7	(56.3–79.6)	0.5850
Fat (g/100 g)	0.0	(0.0–0.0)	0.0	(0.0–0.0)	0.5165
Protein (g/100 g)	0.5	(0.0–0.9)	0.3	(0.0–0.9)	0.1396
Carbohydrates (g/100 g)	15.9	(14.2–17.5)	15.0	(13.3–18.2)	0.2066
Fiber (g/100 g)	1.7	(1.0–2.0)	1.7	(0.9–2.4)	0.8248
Sugar (g/100 g)	12.1	(10.1–13.3)	10.6	(9.7–13.1)	0.0046 *
Sodium (mg/100 g)	0.0	(0.0–5.1)	4.4	(0.0–7.0)	<0.0001 *
Vitamin A (mcg/100 g)	30.3	(0.0–121.2)	0.0	(0.0–106.2)	0.0656
Vitamin C (mcg/100 g)	13.1	(3.4–20.0)	13.9	(9.3–13.9)	0.4502
Calcium (mg/100 g)	0.0	(0.0–10.6)	0.0	(0.0–10.6)	0.8703
Iron (mg/100 g)	0.3	(0.0–0.3)	0.2	(0.0–0.5)	0.7651
Contained added sugar (%) ^1^	47.9%	(38.8–57.2%)	15.0%	(8.8–23.1%)	<0.0001 ^†^
Vegetable-based					
Serving size, g	99.0	(90.0–99.0)	113.0	(71.0–113.0)	0.2108
Calories (Kcal/100 g)	50.5	(35.4–60.6)	48.2	(35.4–65.7)	0.5115
Fat (g/100 g)	0.0	(0.0–0.5)	0.0	(0.0–0.0)	0.4333
Protein (g/100 g)	1.1	(0.6–2.0)	1.0	(0.6–1.7)	0.2086
Carbohydrates (g/100 g)	9.1	(7.1–10.6)	8.8	(7.1–13.3)	0.7368
Fiber (g/100 g)	1.8	(1.0–2.2)	1.7	(1.0–1.9)	0.4319
Sugar (g/100 g)	3.3	(10.3–5.1)	4.0	(2.7–10.0)	0.0489
Sodium (mg/100 g)	26.6	(11.1–53.1)	20.4	(6.5–35.4)	0.1613
Vitamin A (mcg/100 g)	1818.2	(454.5–4545.5)	1460.2	(290.8–4387.2)	0.6154
Vitamin C (mcg/100 g)	0.8	(0.0–2.5)	0.0	(0.0–0.0)	<0.0001 *
Calcium (mg/100 g)	24.2	(14.1–24.2)	21.2	(12.1–30.9)	0.2525
Iron (mg/100 g)	0.4	(0.3–0.6)	0.4	(0.3–0.5)	0.1328
Contained added sugar (%) ^1^	8.1%	(1.7–21.9%)	0.0%	(0.0–5.0%)	0.0143 ^†^

IQR = Interquartile range. ^1^ Percent (%) and 95% confidence interval are presented for containing added sugar. * Statistically significant at q = 0.05 using Benjamini–Hochberg procedure for multiple testing based on 60 comparisons. *p*-value derived from Wilcoxon rank–sum test. ^†^ Statistically significant at alpha = 0.05. *p*-value derived from chi-square test.

## Supplementary Materials

The following are available online at https://www.mdpi.com/2072-6643/11/7/1689/s1, Web Supplement: Product Classification, Table S1: Inclusion and exclusion of products for analytic sample (*n* = 1037), Table S2: Comparison of macro- and micronutrient composition between squeeze pouch and other packaging, including only brands with both squeeze pouch and other products (*n* = 466).

## References

[1] Perez-Escamilla R., Segura-Perez S., Lott M. (2017). Feeding Guidelines for Infants and Young Toddlers: A Responsive Parenting Approach. https://healthyeatingresearch.org/wp-content/uploads/2017/02/her_feeding_guidelines_report_021416-1.pdf.

[2] Cernansky R. (2018). Rethinking Baby Food Pouches. https://www.nytimes.com/2018/06/19/well/rethinking-baby-food-pouches.html.

[3] The Nielsen Company (2015). Global Baby Care Report. http://www.nielsen.com/content/dam/nielsenglobal/de/docs/Nielsen%20Global%20Baby%20Care%20Report%20-%20August%202015.pdf.

[4] ABC Packaging Direct (2017). Baby Food. From Jar to Pouch: The Evolution of Packaging. https://cdn2.hubspot.net/hubfs/70169/reports/BABY%20FOOD%20PACKAGING%20REPORT.pdf?t=1516696274002.

[5] Stasenko N. The Downsides of Baby Food Pouches (And How to Use Them Right). https://www.parents.com/recipes/scoop-on-food/the-downsides-of-baby-food-pouches-and-how-to-use-them-right/.

[6] Children’s Healthcare of Atlanta Strong4Life Are Baby Food Pouches Healthy or Harmful?. https://www.strong4life.com/en/pages/convenience/articles/baby-food-in-a-pouch-healthy-or-harmful.

[7] Koletzko B., Buhrer C., Ensenauer R., Jochum F., Kalhoff H., Lawrenz B., Körner A., Mihatsch W., Rudloff S., Zimmer K.-P. (2019). Complementary foods in baby food pouches: Position statement from the Nutrition Commission of the German Society for Pediatrics and Adolescent Medicine (DGKJ, e.V.). Mol. Cell. Pediatr..

[8] Koletzko B., Lehmann Hirsch N., Jewell J.M., Caroli M., Rodrigues Da Silva Breda J., Weber M. (2018). Pureed Fruit Pouches for Babies: Child Health Under Squeeze. J. Pediatr. Gastroenterol. Nutr..

[9] Theurich M.A. (2018). Perspective: Novel Commercial Packaging and Devices for Complementary Feeding. Adv. Nutr..

[10] Maalouf J., Cogswell M.E., Bates M., Yuan K., Scanlon K.S., Pehrsson P., Gunn J.P., Merritt R.K. (2017). Sodium, sugar, and fat content of complementary infant and toddler foods sold in the United States, 2015. Am. J. Clin. Nutr..

[11] Elliott C.D., Conlon M.J. (2015). Packaged baby and toddler foods: Questions of sugar and sodium. Pediatr. Obes..

[12] Cogswell M.E., Gunn J.P., Yuan K., Park S., Merritt R. (2015). Sodium and sugar in complementary infant and toddler foods sold in the United States. Pediatrics.

[13] Vos M.B., Kaar J.L., Welsh J.A., Horn L.V.V., Feig D.I., Anderson C.A.M., Patel M.J., Munos J.C., Krebs N.F., Xanthakos S.A. (2016). Added Sugars and Cardiovascular Disease Risk in Children: A scientific statement from the American Heart Association. Circulation.

[14] (2017). U.S. Food & Drug Administration. https://www.fda.gov/food/guidanceregulation/guidancedocumentsregulatoryinformation/infantformula/ucm136118.htm.

[15] Benjamini Y., Hochberg Y. (1995). Controlling the false discovery rate: A practical and powerful approach to multiple testing. J. R. Stat. Soc. Ser. B (Methodol.).

[16] Harris G., Coulthard H. (2016). Early Eating Behaviours and Food Acceptance Revisited: Breastfeeding and Introduction of Complementary Foods as Predictive of Food Acceptance. Curr. Obes. Rep..

[17] Blossfeld I., Collins A., Kiely M., Delahunty C. (2007). Texture preferences of 12-month-old infants and the role of early experiences. Food Qual. Prefer..

[18] Northstone K., Emmett P., Nethersole F. (2001). The effect of age of introduction to lumpy solids on foods eaten and reported feeding difficulties at 6 and 15 months. J. Hum. Nutr. Diet. Off. J. Br. Diet. Assoc..

[19] Sakashita R., Inoue N., Kamegai T. (2004). From milk to solids: A reference standard for the transitional eating process in infants and preschool children in Japan. Eur. J. Clin. Nutr..

[20] Food and Drug Administration (U.S. Department of Health and Human Services) (2016). Code of Federal Regulations, 21 CFR 101.9 [Internet]. https://www.gpo.gov/fdsys/pkg/CFR-2016-title21-vol2/pdf/CFR-2016-title21-vol2-part101.pdf.

