# Supplementary material for: Nutrient Content of Squeeze Pouch Foods for Infants and Toddlers Sold in the United States in 2015
Source: Nutrients. 2019 Jul 23;11(7):1689. doi: 10.3390/nu11071689 (PMC6682959; doi:10.3390/nu11071689)
Supplement: Supplementary file 1 [file nutrients-11-01689-s001.pdf]

Web Supplement

Product Classification

We classified the 703 eligible products into five mutually exclusive food categories (protein-based, yogurt- or milk-based, fruits and vegetables, fruit-based, and vegetable-based) based on the full ingredient list of the product. Because many products consisted of mixtures of ingredients, we assigned categories in a hierarchical manner. First, products containing any meat (e.g., beef), fish (e.g., salmon), or legumes (e.g., chickpeas) were assigned to meat-, fish-, or legume-based. If a product did not fall into this category, we continued down the hierarchy of possible food categories. Second, remaining products consisting of yogurts, custards, and puddings were assigned to yogurt- or milk-based. Products containing cheese or butter in a mixture of food categories (e.g., macaroni and cheese with vegetables) were not included in this category in the main analyses. Third, remaining products consisting of a mixture of pureed fruits and vegetables were assigned to fruits and vegetables. Products in this category could also contain grains. Fourth, remaining products consisting of only pureed fruits, or fruits plus grains (e.g., oatmeal), were assigned to fruit-based. Fifth, remaining products consisting of only pureed vegetables, or vegetables plus grains (e.g., pasta with vegetables), were assigned to vegetable-based.

Two authors (JB, MB) independently reviewed the full ingredient list of each product and assigned it a food category. Discrepancies in coding were identified (n = 7) and resolved via discussion. In six cases of discrepancies, one of the authors had incorrectly coded the product due to overlooking a certain ingredient; these instances were corrected by reviewing the product classification scheme. In the final case of discrepancy, a product containing coconut milk and fruit puree was classified by one author as fruit-based and by the other as yogurt- or milk-based. After discussion, the reviewers decided that the product was intended to be a yogurt-based puree, although it was non-dairy due to the inclusion of coconut milk instead of cow's milk.

Table S1. Inclusion and exclusion of products for analytic sample (n = 1,037).

**Products, by packaging type**	**Total, *n***	**Included, *n***	**Excluded, *n***
Total	1037	703	334
Bag products (e.g., crisps, biscuits, puffs, ravioli)	16	0	16
Box products (e.g., meal bowls, cereal packets, cookies, pizza, nutrition bar, rice biscuits, pancakes/waffles, grain-based dinner packets)	89	0	89
Can products (e.g., soup, pasta)	5	0	5
Glass jar products (e.g., pureed ready-to-serve foods, meat sticks, soup). Included all except meat sticks and soup.	216	211	5
Juice box products (e.g., smoothie, juice)	4	0	4
Plastic bottle products (e.g., juice, yogurt drinks)	23	0	23
Plastic bowl products (e.g., hot cereal)	4	0	4
Plastic box products (e.g., teething wafers)	2	0	2
Plastic container products (e.g., cereal, oatmeal, baby cereal, crunchy snacks, crisps, puffs, yogurt, fruit and cereal purees. Yogurts and purees included, all others excluded.	90	19	71

Plastic pack products (e.g., pureed vegetables, pureed mixed dinners, yogurt, pureed fruits). Included all except Lil' Bits products (contain small soft pieces of food intended to help baby learn to chew)	80	71	9
Pouch products (e.g., baby cereal, cereal bars, rice cake, dried vegetables, crackers, twists, cookies, freeze dried yogurt snacks, tiny fruits, add-water meals)	75	5	70
Squeeze pouch products (e.g., pureed, ready-to-serve foods)	397	397	0
Tray products (e.g., prepared meals, fruit pick-ups)	36	0	36

Table S2. Comparison of macro- and micronutrient composition between squeeze pouch and other packaging, including only brands with both squeeze pouch and other products (n = 466).

	Squeeze pouch		Other packaging		
**Nutritional component**	**Median**	**(IQR)**	**Median**	**(IQR)**	**p**
Meat-, fish-, or legume-based					
Serving size, g	99.0	(99.0, 127.0)	113.0	(99.0, 170.0)	0.2214
Calories (Kcal/100 g)	55.1	(50.5, 70.7)	70.7	(61.9, 88.2)	0.0085*
Fat (g/100g)	1.0	(0.0, 2.0)	1.8	(0.9, 3.0)	0.0013*
Protein (g/100g)	3.0	(2.0, 3.1)	2.4	(1.8, 3.2)	0.2905
Carbohydrates (g/100g)	9.1	(8.1, 11.1)	10.1	(8.0, 12.9)	0.6074
Fiber (g/100g)	2.0	(1.6, 2.0)	1.1	(0.6, 1.8)	0.0041*
Sugar (g/100g)	2.4	(1.6, 3.0)	2.9	(1.7, 4.0)	0.3605
Sodium (mg/100g)	15.2	(10.1, 23.6)	29.4	(16.7, 39.8)	0.0260
Vitamin A (mcg/100g)	413.4	(151.5, 681.8)	796.5	(50.0, 1991.2)	0.2059
Vitamin C (mcg/100g)	0.0	(0.0, 0.0)	0.0	(0.0, 0.6)	0.6028
Calcium (mg/100g)	24.2	(12.1, 37.8)	12.4	(10.6, 21.2)	0.0121*
Iron (mg/100g)	0.6	(0.6, 0.9)	0.5	(0.3, 0.7)	0.0279
Contained added sugar (%)[1]	0.0%	(0.0%, 21.8%)	3.2%	(0.4%, 11.0%)	0.4845
Yogurt- or milk-based					
Serving size, g	107.0	(105.0, 120.0)	113.0	(99.0, 113.0)	0.0309
Calories (Kcal/100 g)	88.8	(66.7, 101.0)	97.3	(88.5, 101.0)	0.1087
Fat (g/100g)	1.2	(0.4, 2.0)	3.0	(3.0, 3.5)	<.0001*
Protein (g/100g)	1.9	(0.8, 2.0)	3.0	(3.0, 3.5)	0.0002*
Carbohydrates (g/100g)	16.3	(14.2, 18.3)	14.7	(12.4, 16.2)	0.0271
Fiber (g/100g)	0.8	(0.0, 1.2)	0.0	(0.0, 0.4)	0.001*
Sugar (g/100g)	12.3	(11.4, 13.3)	11.5	(10.6, 12.1)	0.0057*
Sodium (mg/100g)	25.0	(12.5, 35.4)	53.1	(47.4, 55.6)	<.0001*
Vitamin A (mcg/100g)	79.2	(30.3, 166.7)	213.2	(132.7, 239.9)	0.0013*
Vitamin C (mcg/100g)	10.0	(0.7, 20.0)	0.0	(0.0, 0.0)	<.0001*
Calcium (mg/100g)	90.9	(59.8, 152.4)	106.2	(90.9, 132.7)	0.8424
Iron (mg/100g)	0.1	(0.0, 0.3)	0.0	(0.0, 0.0)	0.0088*
Contained added sugar (%)[1]	73.5%	(55.6%, 87.1%)	78.6%	(59.1%, 91.7%)	0.6446
Fruits and vegetables					
Serving size, g	113.0	(99.0, 113.0)	60.0	(60.0, 113.0)	<.0001*
Calories (Kcal/100 g)	59.5	(50.3, 66.0)	58.3	(50.0, 64.3)	0.3218
Fat (g/100g)	0.0	(0.0, 0.0)	0.0	(0.0, 0.0)	0.2429

Protein (g/100g)	0.4	(0.0, 0.9)	0.0	(0.0, 0.4)	0.0033*
Carbohydrates (g/100g)	13.3	(12.1, 15.0)	13.3	(11.4, 13.3)	0.2432
Fiber (g/100g)	1.8	(0.9, 2.0)	1.7	(1.7, 2.5)	0.3592
Sugar (g/100g)	10.0	(7.5, 10.8)	8.3	(6.7, 10.0)	0.0391
Sodium (mg/100g)	9.1	(4.4, 17.2)	2.2	(0.0, 16.7)	0.0192
Vitamin A (mcg/100g)	353.4	(123.1, 1051.1)	615.5	(50.0, 1413.7)	0.6702
Vitamin C (mcg/100g)	11.2	(0.0, 15.9)	0.0	(0.0, 1.0)	<.0001*
Calcium (mg/100g)	10.6	(0.0, 12.1)	20.0	(5.0, 20.0)	0.0055*
Iron (mg/100g)	0.3	(0.0, 0.5)	0.5	(0.3, 0.5)	0.0690
Contained added sugar (%)[1]	31.8%	(18.6%, 47.6%)	33.3%	(18.6%, 51.0%)	0.8855
Fruit-based					
Serving size, g	99.0	(99.0, 113.0)	113.0	(99.0, 113.0)	0.4196
Calories (Kcal/100 g)	66.7	(58.3, 80.8)	64.7	(56.3, 79.6)	0.7962
Fat (g/100g)	0.0	(0.0, 0.0)	0.0	(0.0, 0.0)	0.6177
Protein (g/100g)	0.5	(0.4, 0.9)	0.3	(0.0, 0.9)	0.0450
Carbohydrates (g/100g)	15.6	(13.3, 17.7)	15.0	(13.3, 18.2)	0.5178
Fiber (g/100g)	1.7	(1.0, 2.0)	1.7	(0.9, 2.4)	0.8080
Sugar (g/100g)	11.9	(10.8, 12.9)	10.6	(9.7, 13.1)	0.0082*
Sodium (mg/100g)	4.2	(0.0, 5.1)	4.4	(0.0, 7.0)	0.0709
Vitamin A (mcg/100g)	48.0	(0.0, 202.0)	0.0	(0.0, 106.2)	0.0269
Vitamin C (mcg/100g)	13.9	(4.0, 18.2)	13.9	(9.3, 13.9)	0.4437
Calcium (mg/100g)	0.0	(0.0, 10.6)	0.0	(0.0, 10.6)	0.3654
Iron (mg/100g)	0.3	(0.0, 0.3)	0.2	(0.0, 0.5)	0.7522
Contained added sugar (%)[1]	29.6%	(18.0%, 43.6%)	15.0%	(8.8%, 23.1%)	0.0276†
Vegetable-based					
Serving size, g	99.0	(99.0, 99.0)	113.0	(71.0, 113.0)	0.3068
Calories (Kcal/100 g)	45.5	(35.4, 60.6)	48.2	(35.4, 65.7)	0.3957
Fat (g/100g)	0.0	(0.0, 0.5)	0.0	(0.0, 0.0)	0.3976
Protein (g/100g)	1.0	(0.5, 1.1)	1.0	(0.6, 1.7)	0.9706
Carbohydrates (g/100g)	8.1	(6.7, 9.4)	8.8	(7.1, 13.3)	0.1891
Fiber (g/100g)	1.0	(1.0, 1.1)	1.7	(1.0, 1.9)	0.0134*
Sugar (g/100g)	4.4	(2.0, 5.1)	4.0	(2.7, 5.8)	0.6644
Sodium (mg/100g)	20.2	(15.2, 55.6)	20.4	(6.5, 35.4)	0.1827
Vitamin A (mcg/100g)	378.8	(378.8, 1818.2)	1460.2	(290.8, 4387.2)	0.1269
Vitamin C (mcg/100g)	0.0	(0.0, 15.9)	0.0	(0.0, 0.0)	0.0331
Calcium (mg/100g)	24.2	(12.1, 26.7)	21.2	(12.1, 30.9)	0.6457
Iron (mg/100g)	0.3	(0.2, 0.3)	0.4	(0.3, 0.5)	0.4564
Contained added sugar (%)[1]	0.0%	(0.0%, 5.0%)	0.0%	(0.0%, 24.7%)	N/A‡

IQR = Interquartile range. [1] Percent (%) and 95% confidence interval are presented for containing added sugar. * Statistically significant at q = 0.05 using Benjamini-Hochberg procedure for multiple testing based on 60 comparisons. p-value derived from Wilcoxon Rank-Sum test. † Statistically significant at alpha = 0.05. p-value derived from chi-square test. ‡ Chi-square test not conducted because no vegetable-based products contained added sugars.
